# A Rare Case of Hepatic Artery Vasospasm in a Native Liver

**DOI:** 10.7759/cureus.93219

**Published:** 2025-09-25

**Authors:** Chelsea Oduol, Ayomide Olusina, Alixis Dumorney, Tamara Harrell, Kyle Farrow, Stephen Symes

**Affiliations:** 1 Internal Medicine, St. George's University School of Medicine, St. George's, GRD; 2 Internal Medicine, Ross University School of Medicine, St. Michael, BRB; 3 Infectious Diseases, University of Miami Miller School of Medicine, Miami, USA

**Keywords:** calcium channel blockers, hepatic artery vasospasm, homocysteine, liver enzymes, liver transplant

## Abstract

Hepatic artery vasospasm (HAV) is a relatively uncommon vascular phenomenon that can arise after liver transplants. Its occurrence in a native liver presents an atypical clinical picture. There is a lack of suﬃcient data on its etiology, incidence, diagnostics and management leaving room for further investigation. This report presents a rare case of HAV in a 40-year-old woman with a native liver. A recent hip fall led to her admission, followed by markedly elevated liver enzymes one week later. Doppler ultrasound (US) revealed patent hepatic vasculature with an elevated resistivity index, which supported our presumed diagnosis. Because of limited data on preventative management for this diagnosis, we used the management of vasospastic angina to start a treatment course, which included a calcium channel blocker and a Statin. Her liver enzymes began to normalize, indicating a favorable response to treatment, ultimately leading to discharge.

## Introduction

Hepatic artery vasospasm (HAV) is a state of sudden arterial narrowing causing decreased blood supply to the liver [1]. Clinically, it results in elevated liver enzymes presenting similarly to hepatic artery thrombosis (HAT) and stenosis [1]. There are no recorded cases of HAV in patients without a history of a liver transplant. In this specific case, we performed many studies to determine the root cause of the elevated liver enzymes, aspartate aminotransferase (AST) and alanine aminotransferase (ALT). Although there is a lack of standard diagnostic criteria for HAV, studies use Doppler US to diﬀerentiate it from HAT and stenosis [2]. Our patient had an abnormal Doppler US with subsequent ultrasounds showing normal findings, supporting the transient nature of vasospasm and injury. Based on the patient’s clinical course, our report proposes a hypothesis regarding the etiology of the vasospasms and potential preventative strategies.

## Case presentation

A 40-year-old woman came to the emergency department on 11/11/23 with left hip pain after a recent fall. She had developed abdominal pain and elevated liver enzymes one week into her admission.

Past history includes 2/2 positive MTHFR hypercoagulable state secondary to hyperhomocysteinemia (with levels of homocysteine ranging from 6.45 to >15, normal: <10.4 umol/L). There were multiple pulmonary embolisms and deep vein thrombosis with a positive lupus anticoagulant. She has had a transient ischemic attack and a cerebrovascular accident, untreated with statins due to a previous allergy. She suffers from fibromyalgia with chronic pain syndrome, migraines, asthma, and hypertension, and paroxysmal atrial fibrillation with multiple ablations. She has had multiple admissions for right upper quadrant pain, with studies for viral, autoimmune, and genetic causes of hepatitis, as well as evaluations for primary biliary cirrhosis, C1 esterase deficiency, and lupus (anti-smooth muscle and anti-dsDNA), all yielding negative results.

Surgical and procedural history includes endoscopic retrograde cholangiopancreatography for biliary stenosis with chronic itching, and sphincterotomy for presumed Sphincter of Oddi dysfunction. She had a left hip replacement on 7/20/23, secondary to bilateral hip avascular necrosis. She has had multiple ablations for paroxysmal atrial fibrillation.

Clinical findings and timeline

The patient developed nausea, vomiting, and abdominal pain, with clinical findings of right upper quadrant tenderness and no icterus or hepatomegaly.

**Figure 1:**
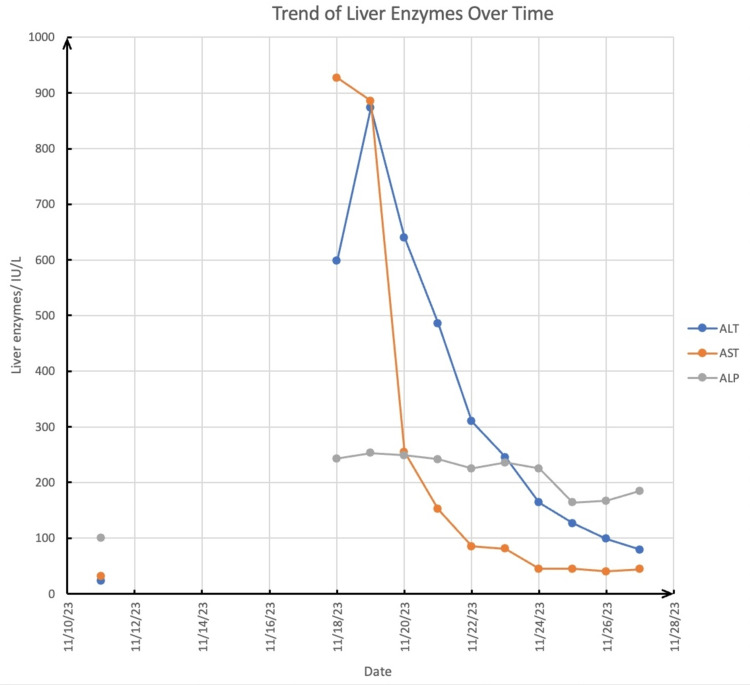
Trend of Liver Enzymes Over Time

Information in this section refers to Figure 1, the graph of liver enzymes plotted against time, unless stated otherwise. On admission (11/11/23), her liver enzymes were normal (ref. ALT 9-45, AST 17-59, ALP 38-126) but rapidly increased five days later to ALT 598 IU/L, AST 927 IU/L, and ALP 243 IU/L. Her initial aPTT of 119 (not depicted in Figure 1) was high and remained elevated throughout her hospitalization on heparin, followed by Coumadin.

**Figure 2:**
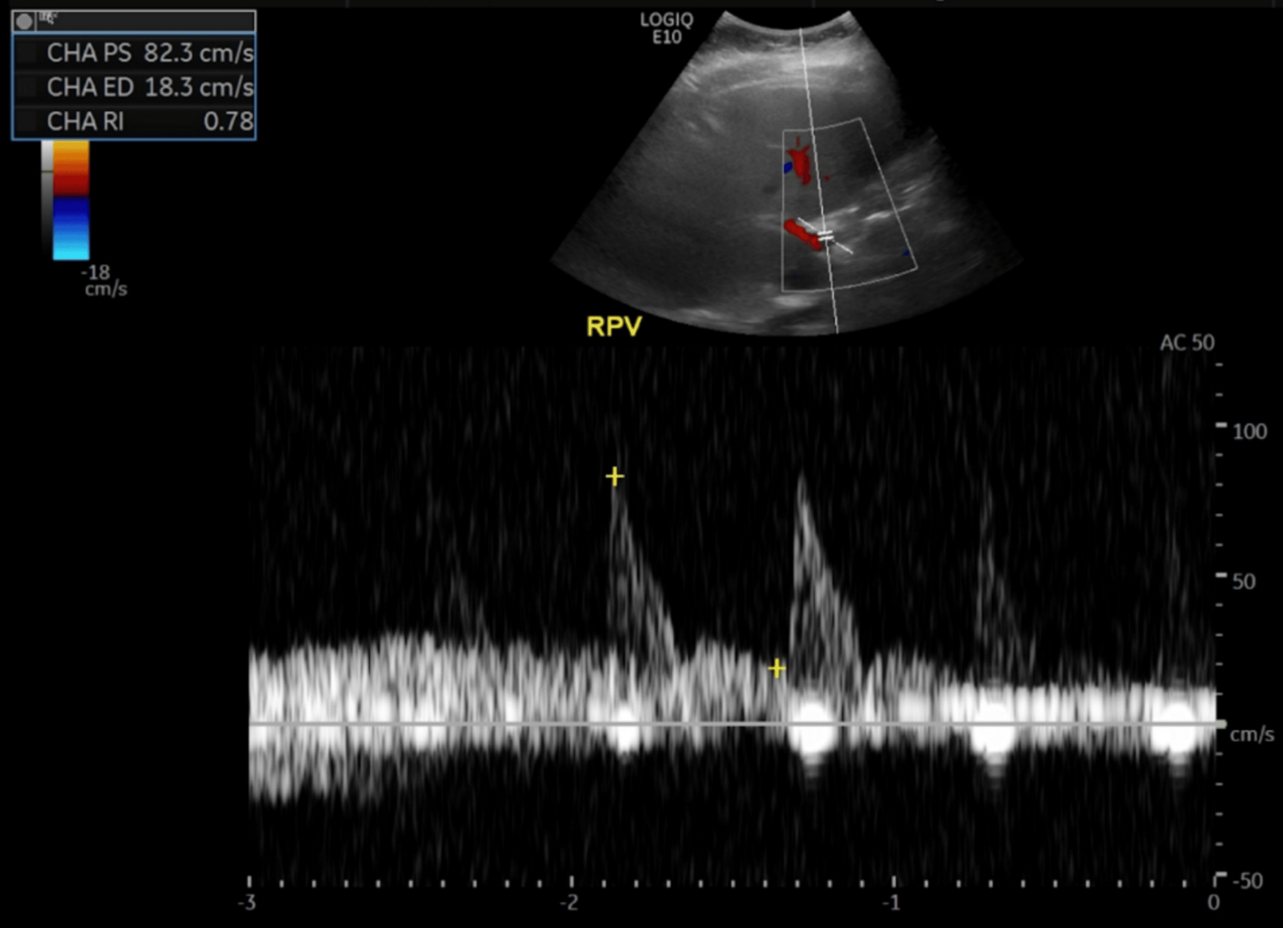
Doppler Ultrasound of the Liver

On 11/18/23, comprehensive toxicology screening, urine porphobilinogen, and genetic testing for acute hepatic porphyria, including ferrochelatase and aminolevulinic acid synthase (ALAS2) sequencing, were unremarkable. Ultimately, she had three liver biopsies that yielded no specific etiology, despite marked elevations in liver enzymes during one biopsy. She experienced severe constipation, abdominal bloating, and distension with minimal improvement on MiraLAX, GoLYTELY 1000mL, Lactulose, and Soapsuds enemas. Serial abdominal radiographs consistently demonstrated non-obstructive bowel gas patterns but moderate stool burden in the ascending and transverse colon. Doppler US of the liver revealed patent hepatic vasculature without biliary ductal dilation, appropriate anterograde flow in the common hepatic artery with a peak systolic velocity of 82.3 cm/s, and an elevated resistivity index (RI) of 18.3 (normal: 0.55-0.8). The elevated RI (18.3) supported a presumptive diagnosis of HAV (Figure 2) [3].

We started 240 mg oral Diltiazem (11-21-23), with a therapeutic goal of 480 mg depending on tolerance. Overall, we noted a consistent downtrend in liver enzymes from 11/21/23-11/27/23, with the greatest decrease in ALT from 486 IU/L to 79 IU/L, followed by AST from 152 IU/L to 44 IU/L and ALP from 242 IU/L to 185 IU/L.

Although the patient presented on 11/11/23, it is important to indicate findings throughout the prior five years and results of the diagnostic workup during that period. On July 23, 2018, an episode of epigastric pain with vomiting prompted a CT-guided liver biopsy, which showed minimal non-specific changes. Liver enzymes were elevated with AST of 137, ALT of 434, and ALP of 219. The following day, AST was 116, ALT 347, and ALP of 231, with a negative work-up for autoimmune hepatitis. Advancing to February 2023, similar symptoms brought the patient in for evaluation, with again hepatic biopsy changes that were mild and non-specific. The eight portal tracts in the sample contained a preserved interlobular bile duct. The portal tracts were uninflamed, and there was no interface hepatitis. Mild sinusoidal dilation in the lobules was accompanied by areas of atrophy and regeneration. Mild steatosis (<5%) was present. Hepatic workup, a few days later, showed an AST of 47, ALT of 158, and ALP of 234. At this time, viral serologies were negative, as were anti-nuclear antibodies (ANA), antimitochondrial antibodies (AMA), and anti-smooth muscle antibodies (ASMA). Overall, the changes seen were non-specific, suggestive of resolving/ regenerative changes related to prior hepatic injury. Similar symptoms prompted admission in June 2023, with a Duplex U/S, on June 29, 2023, of the liver with abdominal U/S demonstrating patent hepatic vessels as well as an RI of 0.7 (normal: 0.55-0.8). Labs showed once again elevated AST 627, ALT 737, and ALP 224. These numerous US studies and biopsies were due to the patient’s intermittent and unresolved right upper quadrant abdominal pain with unexplained transaminitis.

## Discussion

Diagnostic challenges

We had trouble choosing a suitable diagnostic test because all past studies were unremarkable, including a biopsy done during the time her AST/ALT was elevated. Biopsy results yielded negative, possibly from HAVs not being profound enough, nor duration long enough, to cause significant changes in liver pathology. In ischemic hepatitis [4], we see changes in biopsy due to zone 3 being prone to necrosis because of its lower oxygenation levels compared to zones 1 and 2 of the liver. The transient nature of her symptoms made getting labs and testing unpredictable and diﬃcult. We chose to focus the investigation on the liver, considering the patient’s complex past history, including a hypercoagulable state. Finally, HAV is typically seen in the setting of a liver transplant, which the patient did not have.

Therapeutic interventions

She received several pharmacological agents, which included heparin 25,000 units as a bridging therapy to coumadin 12.5 mg, Benadryl 25mg I.V. PRN, metoprolol 50mg, and diltiazem 240mg, to be increased to 480mg if tolerated. Coumadin, metoprolol, and diltiazem were given orally. The patient was switched from heparin to Coumadin after the bridge was completed.

Assessment and follow-up

The presumptive diagnosis was transient HAV leading to shock liver since multiple liver biopsies came back negative. These vasospastic episodes are hypothesized to be from direct endothelial injury from hyperhomocysteinemia [5,6]. The patient is positive for lupus anticoagulant and hypercoagulable state and has methylenetetrahydrofolate reductase deficiency leading to hyperhomocysteinemia. She was started on diltiazem 240 mg without experiencing lower extremity edema. We planned to monitor and gradually increase to a final dose of 480mg. Upon discharge, the patient's liver enzymes were improving.

Discussion

In this section, we explore a possible etiology for HAV based on the patient's medical history, response to treatment, and diagnostic limitations. The patient’s extensive medical history seems to revolve around her hypercoagulable state-MTHFR deficiency, resulting in hyperhomocysteinemia. She had multiple admissions for right upper quadrant abdominal pain associated with elevated liver enzymes of an undetermined cause. Doppler US showed an elevated RI, suggesting HAV [3], playing a pivotal role in the search for an etiology and likely treatment. Other than limiting the use of hepatotoxic drugs and monitoring her liver enzymes daily, the US results tailored our approach. To prevent future episodes of HAVs, we discussed the trial use of nifedipine, a dihydropyridine calcium channel blocker, as it reduces HAVs post-liver transplant [3]. We avoided nifedipine, since it is associated with a higher risk of lower extremity edema than Diltiazem, and our patient previously experienced lower extremity edema on diltiazem. We viewed the pathogenesis behind coronary artery vasospasms (CAVs) and applied similar treatments. CAVs are postulated to result from endothelial dysfunction that can manifest as impaired endothelial relaxation or increased endothelial excitability [7]. The first line of treatment for CAVs is calcium channel blockers (CCBs) [8]. Calcium is a potent mediator for vascular smooth muscle contraction, leading to impaired relaxation; therefore, we incorporated Calcium channel blockers (Diltiazem 240mg) into her treatment regimen for HAVs.

After beginning treatment with diltiazem, her liver enzymes showed a downtrend (Figure 1), suggesting a therapeutic eﬀect. We recognize the improvement could also reflect a reduction in opioids prescribed for pain. Opioids are hepatotoxic in large amounts, i.e., overdose or as an idiosyncratic response [9]. The patient’s negative expanded toxicology screen makes other drug-induced hepatotoxicity unlikely. Our case is noteworthy as the only documented one with HAVs not instigated by an early complication of liver transplant. Existing studies [10] use Duplex US of the common hepatic artery after liver transplantation to rule out thrombosis and stenosis of the hepatic artery and measure the RI. When the RI is elevated (greater than 0.8), it confirms the diagnosis of HAV.

Our patient’s elevated RI (Figure 2) is comparable to the RIs of the existing studies [3,11] on HAVs; however, she does not have the same predisposing factor (post-liver transplantation). Given her episodic elevations in liver enzymes, hypercoagulable state, and other negative medical workup, it is probable she experienced episodes of HAVs leading to transient ischemic hepatitis. In brief, HAVs in a native liver are a novel finding beyond existing reports.

**Figure 3:**
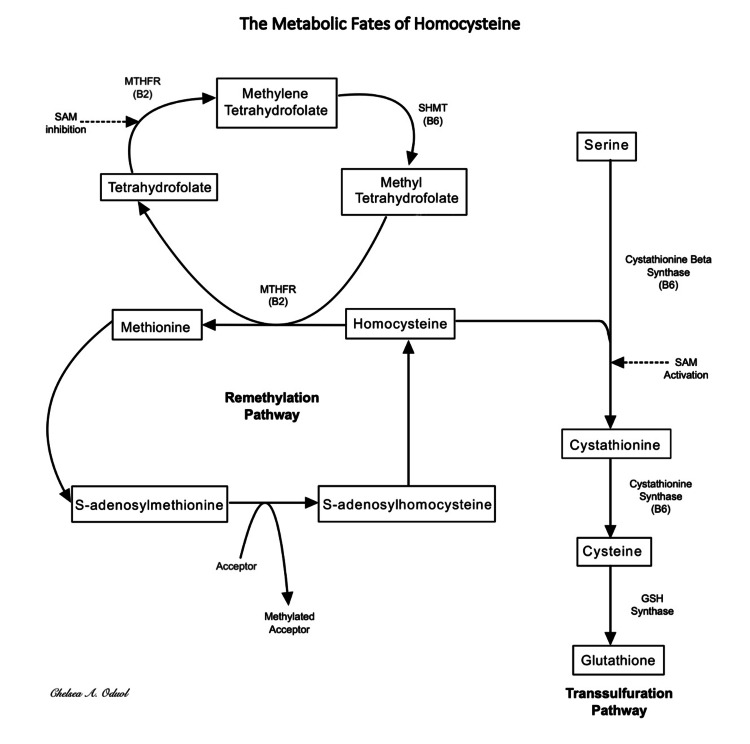
Metabolism of Homocysteine Flow chart depicting the 2 metabolic fates of homocysteine, i.e., transsulfuration and remethylation. The flow chart was constructed using Microsoft Word for the text and notability for the arrows

After extensive research on the possible etiologies of vasospasm, we postulated a hypothesis specific to our patient’s medical history, hepatic vasospasm as a result of direct endothelial injury from homocysteine [5,6]. Our patient has a confirmed hyperhomocysteinemia 2/2 MTHFR deficiency, which leaves excess amounts of homocysteine for metabolism through the cystathionine synthase pathway (Figure 3).

Blood vessels are inherently deficient in this enzyme [6], leading to the accumulation of homocysteine and an increased likelihood of endothelial injury. Direct endothelial injury results in the endothelial cells generating fewer amounts of endogenous nitric oxide [6], a potent vasodilator, increasing the vasospasm probability. Though no studies directly link hyperhomocysteinemia to HAV, there are studies that link elevated homocysteine to endothelial dysfunction, which is critical in the pathogenesis of vasospasms.

## Conclusions

Here, we report the first documented case of HAV in a patient with a native liver. The etiology, risk factors, and clinical course for HAV should be investigated further and included as a diﬀerential in those presenting with elevated liver enzymes without an identifiable cause. Though there is limited understanding of the role liver transplantation has, investigating the existing conditions of the patient, including homocystinuria and iatrogenic hepatic insults from previous liver biopsies, can serve as possible risk factors for the disease. Investigations on treatment alternatives for patients who cannot tolerate CCBs, or possible vitamin B12 supplementation in patients with MTHFR deficiency, could reduce the recurrence of future vasospastic events. Although there is limited literature on the disease and treatment, our patient’s condition continues to improve with Diltiazem 480 mg.

This case may provide valuable insights into recognizing HAVs in patients with native livers, based on our knowledge of the behavior and management of HAVs in transplanted livers, ultimately enhancing clinical decision-making.
